# Supplementary material for: Heterogeneous survival upon disinfection underlies evolution of increased tolerance
Source: Microbiol Spectr. 2024 Oct 22;12(12):e03276-22. doi: 10.1128/spectrum.03276-22 (PMC11619369; doi:10.1128/spectrum.03276-22)
Supplement: Supplemental material — Fig. S1 to S3; Table S1; Methods. [file spectrum.03276-22-s0001.pdf]

Supplementary Information

Heterogeneous survival upon disinfection underlies evolution of increased tolerance

Niclas Nordholt[a], Lydia-Yasmin Sobisch[a], Annett Gödt[a], Dominique Lewerenz[a], Frank Schreiber[a]

[a]Division of Biodeterioration and Reference Organisms (4.1), Department of Materials and the Environment, Federal Institute for Materials Research and Testing (BAM), Berlin, Germany

Contents:

Supplementary methods (Text S1, S2, S3)

Supplementary figures (Figures S1, S2, S3)

Supplementary tables (Tables S1)

Supplementary references

Supplementary methods

Text S1. Model fitting algorithm

To avoid fit instability caused by the correlation of parameters *d* and *p* [1], [2], an initial fit was done using the 'differential evolution' method with randomized initial parameters to approximate the globally optimal parameters. The best set of parameters served as starting point for the 'Nelder-Mead' minimization algorithm. If fitting instability was still observed, the complexity of the model was reduced by setting N_0 to the average of the measured cfu at time point zero. The fits of equations (2) and (3) to the data were ranked based on the AIC corrected for small sample size (AICc), which takes into account the goodness of fit and the parsimony of the model [3]. The robustness of the fitting method to measurement noise was assessed in-depth (Figure S3, Text S2). The following data points were not considered for model fits: zero counts due to unknown uncertainty in those measurements. Data points which increased more than 3-fold in comparison to the previous timepoint (6 of 316 non-zero data points [1.9%]) and data with only a single replicate measurement (2 of 316 non-zero data points [0.63%]) where not considered due to high chance of resulting from experimental errors.

Text S2. Derivation of Weibull model for chemical inactivation

Killing kinetics are often interpreted as first-order mortality kinetics, i.e. exponential decay at a fixed rate. Alternatively, they can be seen as the cumulative function of the distribution of tolerance times of a bacterial population, where the survival of an individual cell is determined by its tolerance phenotype, i.e. the time for which it can survive a lethal stress [2], [4], [5]. This interpretation appreciates the phenotypic heterogeneity in bacterial populations. It also allows to model killing kinetics that are not well fitted by first-order kinetics, without the need to invoke ad-hoc parameters to explain frequently observed deviations from log-linearity, such as downward or upward curvatures (Figure S2). To model the time-kill kinetics of *E. coli* exposed to lethal concentrations of chlorhexidine (CHX), benzalkonium chloride (BAC), 2-propanol (ISO), didecyldimethylammonium chloride (DDAC), hydrogen peroxide (H_2O_2) and glutaraldehyde (GTA), we considered a model based on the cumulative survival function of the sum of two mixed Weibull distributions [2]. The Weibull distribution can fit a wide range of kinetics due to its flexibility provided by its shape parameter. The two distributions describe the tolerance times of two populations, i.e. the major, susceptible population and a minor, more tolerant persister subpopulation. The model describes the number of survivors *N* consisting of the two subpopulations as function of time *t* as:

$$N(t) = \frac{N_0}{1 + 10^a}\left(10^{-\left(\frac{t}{d_1}\right)^p + a} + 10^{-\left(\frac{t}{d_2}\right)^p}\right) \tag{S1}$$

where N_0 is the inoculum size in cfu/mL, a is the logit transformation of the initial fraction f of the susceptible population 1 ($a = \log\frac{f}{1-f}$), p is a shape parameter and d_1 and d_2 are parameters associated to the tolerance of the populations. Specifically, they are the treatment times for the first decimal reduction of population 1 (susceptible) and 2 (persister), respectively. We use the simplified model by Coroller *et al.* which sets the shape parameter p to be the same for both Weibull distributions [2]. The shape parameter p is related to the heterogeneity in the population via the coefficient of variation (CV = standard deviation / mean) as follows: if p = 1, CV = 1; if p > 1, CV < 1; and if p < 1, CV > 1. By setting p = 1, the model simplifies to the well-known model commonly used in antibiotic persistence research [6], which can also be interpreted as the sum of two exponential distributions:

$$N(t) = \frac{N_0}{1 + 10^a}\left(10^{\frac{k_1 t}{\ln(10)} + a} + 10^{\frac{k_2 t}{\ln(10)}}\right) = N_0\left(fe^{-k_1 t} + (1-f)e^{-k_2 t}\right) \tag{S2}$$

where k_1 and k_2 are the rate parameters at which each subpopulation dies, with $k_i = \frac{\ln(10)}{d_i}$. When only one population is present, i.e. fraction f = 1, the bimodal equations (2) and (3) simplify to unimodal equations:

$$N(t) = N_0 10^{-\left(\frac{t}{d_1}\right)^p} \tag{S3}$$

and with p = 1:

$$N(t) = N_0 10^{-\left(\frac{t}{d}\right)} = N_0 e^{-kt} \tag{S4}$$

Equation (S1), and its special cases equations (S2) – (S4), can be used to fit a wide range of inactivation kinetics, including those that deviate from the idealistic log-linear decrease, as exemplified in Figure S2. The tolerance time distributions obtained from time kill kinetics are straightforwardly used to sample survival times for statistical modelling approaches [7].

Text S3. Robustness of the fitting algorithm to sample variability and parameter dependence

To assess the susceptibility of the fitting algorithm to variation between replicates, synthetic datasets with different levels of variability were generated and fitted with the algorithm. The data was generated from the uni- or bimodal model (equation (2) and (3)) with errors sampled from a log-normal distribution with mean 0 and standard deviation ranging from 0 – 0.6 in steps of 0.05, resulting in average coefficients of variation (CV) of the data between 0.12 – 2.24 (Figure S3 A). For each CV, 15 datasets with each 6 replicates were generated and fitted by the algorithm. The number of successes in identifying the correct model were then tested against the null hypothesis that model selection is random, i.e. the chance to pick the correct model is 50% or lower, using a binomial test. Under the given assumptions, significance to a 95% level ($p < 0.05$) is achieved when the correct model is chosen in at least 12/15 cases. The average CV per condition over all time points in our experimental data ranged from 0.22 – 1.05, which is well within the range where the algorithm reliably selects the correct model (Figure S3 A).

An important property of Weibull distributions is the dependence between the parameters p and d_1 in equations (S1)/(1) and (S3)/(3), which can result in instabilities when determining the correct parameter values [1], [2]. For most of our data this was not the case, and the optimal values could be determined by the algorithm reliably. We assessed the ability of the algorithm to identify the optimal values of p and d_1 by fixing one of the parameters while varying the value of the other and comparing the goodness of fit via the AIC for the dataset with the highest variation between replicates, CHX (Figure 1 C). The algorithm was able to identify the parameter value which resulted in the best fit of the model to the data as determined by the minimal χ^2 (Figure S3 B, C) with reasonable parameter uncertainties (Table S1).

Supplementary figures

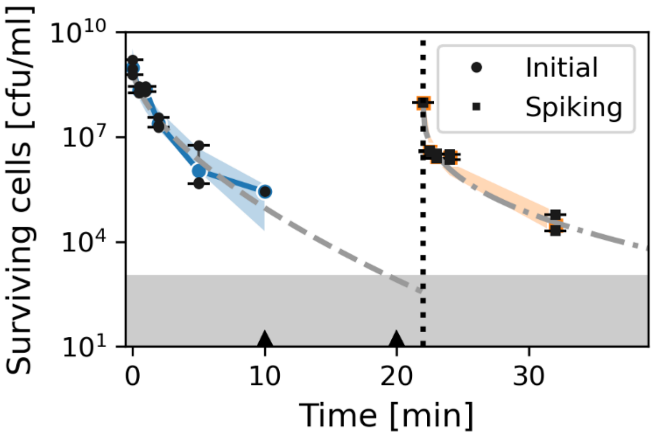

Figure S1. Prolonged time-kill kinetics of chlorhexidine are not caused by exhaustion of disinfectant from the medium. Biological activity of chlorhexidine is maintained beyond a 20-minute time-kill assay, showing that the time-kill kinetics are not caused by exhaustion of the substance. The vertical dotted line indicates the time when cells from the original culture were spiked into the killing assay. Number of biological replicates n = 3. Blue and orange symbols indicate the geometric mean, error bars indicate the 95% C.I. obtained by bootstrapping. Small grey symbols are datapoints of individual experiments. Black triangles on the x-axis indicate when zero-counts were present. The dashed line and the dash-dotted lines are fits of single Weibull distributions to the data. The shaded areas around the fits indicate the 95 % C.I. of the model fit, excluding values with zero counts. The grey shaded area at the bottom indicates the detection limit.

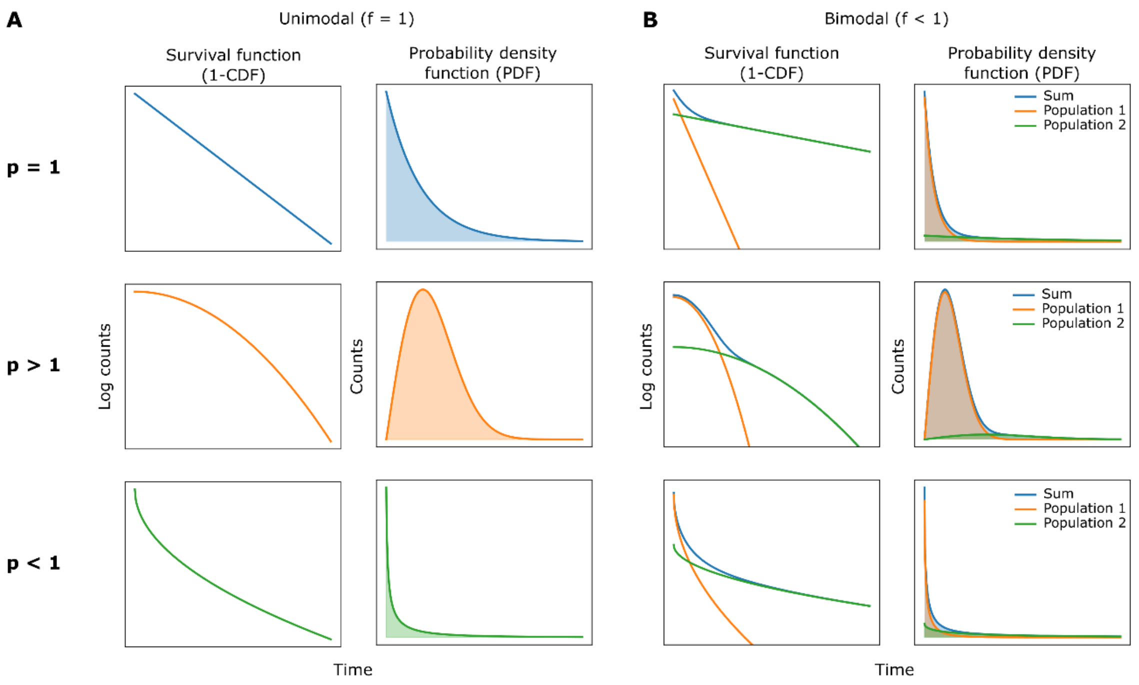

Figure S2. Schematic depiction of different inactivation kinetics as generated by Weibull distributions. Disinfection kinetics can be interpreted as the survival function (left panel) of a Weibull distribution, which can be represented as the probability density function (right panel), highlighting the distribution of individual tolerance times within the bacterial population. **(A)** The shape and the spread of the tolerance time distributions depends on the shape parameter *p*. For the unimodal case $p = 1$ corresponds to a coefficient of variation (CV) = 1; $p > 1$ corresponds to a CV < 1; and $p < 1$ corresponds to a CV > 1. **(B)** Examples of bimodal distributions indicating the presence of two populations, i.e. the fraction *f* of susceptible cells is smaller than 1. The bimodal distribution (blue line) is the sum of two unimodal distributions (orange and green lines) with fractions *f* and 1-*f*, according to equation (2). The shape parameter *p* is the same for the distributions of population 1 and 2, as in [2]. CDF, cumulative distribution function; PDF, probability density function.

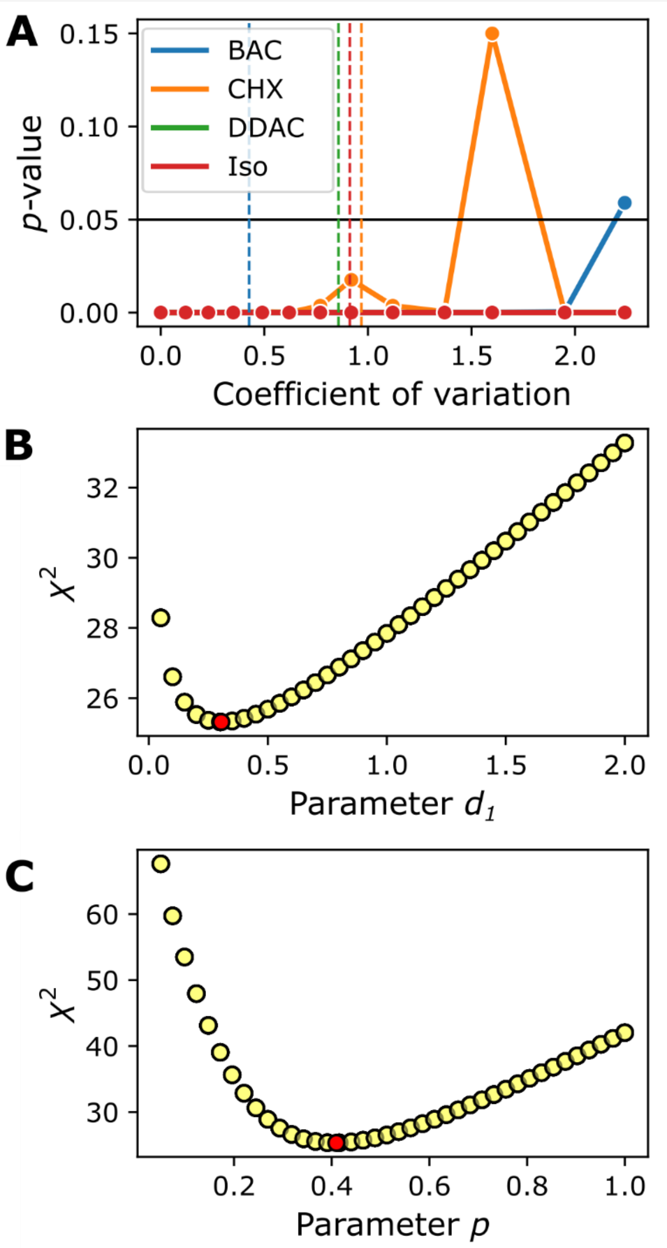

Figure S3: The fitting algorithm robustly identifies the correct model and the optimal value for correlated parameters d_1 and *p*. (A) Coefficient of variation (CV) in synthetic data versus the p-value of the algorithm to be better than chance at selecting the correct model (see methods for details). The black line indicates a *p*-value of 0.05. Colored dashed lines indicate the average CV per time point for different experiments in Figure 1 in the main text. **(B, C)** The fitting algorithm is able to identify the optimal values for the correlated parameters d_1 and *p*. The parameters **(B)** d_1 and **(C)** *p* were fixed to different values and the best fit was computed. The goodness of fit (χ^2) is plotted versus the value to which the parameter was fixed (yellow symbols). The best fit value from Table S1, when both parameters were varied freely, is indicated in red.

Supplementary tables

Table S1. Parameter values of the best fits of the Weibull distribution to the data. Errors indicate the standard error of the estimated parameters.

	**Model parameters**							
**Substance**	***N_0***[a] **[cfu/mL]**	***P***[b]	***d1***[c] **[min]**	***d2***[d] **[min]**	***a***[e]	**Best fit**[f]	**AIC weight**[g] **(evidence ratio**[h]**)**	**Application concentration (from [8])**
Hydrogen peroxide (H_2O_2)	1.31 ± 0.16 × 10^9	3.03 ± 0.24	2.60 ± 0.09	n.a.	n.a.	Single Weibull	0.84 (5.16)	7.35 – 10290 mM
Glutaraldehyde (GTA)	9.21 ± 1.27 × 10^8	1.76 ± 0.1	0.90 ± 0.05	n.a.	n.a.	Single Weibull	0.935 (14. 46)	0.0025 – 2 %
Chlorhexidine (CHX)	1.8 ± 1.04 × 10^9	0.41 ± 0.06	0.3 ± 0.17	n.a.	n.a.	Single Weibull	0.72 (2.57)	500 – 20000 µg/mL
Benzalkonium chloride (BAC)	5.16 ± 1.88 × 10^8	1.03 ± 0.20	2.52 ± 0.44	14.99 ± 3.72	2.52 ± 0.29	Double Weibull	0.9998 (4783.97)	60 – 20000 µg/mL
Didecyl-dimethyl-ammonium chloride (DDAC)	3.02 ± 0.85 × 10^8	1.35 ± 0.16	3.32 ± 0.4	13.69 ± 4.65	3.87 ± 0.58	Double Weibull	0.999 (65659.64)	250 – 5000 µg/mL
Isopropanol (ISO)	1.47 ± 0.51 × 10^9	1.33 ± 0.21	1.68 ± 0.27	4.45 ± 0.98	3.12 ± 0.48	Double Weibull	0.996 (257.3)	70 %

Parameters from equation (2), where [a]N_0 is the initial cell density, [b]*p* is the shape parameter, [c]*d1* and [d]*d2* are the duration until first decadic reduction of the susceptible and persister population, respectively, and [e]*a* is the logit transformation of the initial fraction of the susceptible population. n.a., not applicable. [f] Best fit either by equation (2) with Double Weibull or equation (3) with Single Weibull. [g] The AIC weight gives the probability of a model to be the best out of all tested models. [h] The evidence ratio of the best model over the second best model. The evidence ratio gives the relative likelihood, i.e. how much more likely the best model is over the second best model.

Supplementary references

[1] P. Mafart, O. Couvert, S. Gaillard, and I. Leguerinel, “On calculating sterility in thermal preservation methods: application of the Weibull frequency distribution model,” *International Journal of Food Microbiology*, vol. 72, no. 1, pp. 107–113, Jan. 2002, doi: 10.1016/S0168-1605(01)00624-9.

[2] L. Coroller, I. Leguerinel, E. Mettler, N. Savy, and P. Mafart, “General Model, Based on Two Mixed Weibull Distributions of Bacterial Resistance, for Describing Various Shapes of Inactivation Curves,” *Appl. Environ. Microbiol.*, vol. 72, no. 10, pp. 6493–6502, Oct. 2006, doi: 10.1128/AEM.00876-06.

[3] K. P. Burnham and D. R. Anderson, *Model Selection and Multimodel Inference: A Practical Information-Theoretic Approach (2nd ed)*, vol. 172. 2002. [Online]. Available: http://linkinghub.elsevier.com/retrieve/pii/S0304380003004526

[4] M. Peleg and M. B. Cole, “Reinterpretation of Microbial Survival Curves,” *Critical Reviews in Food Science and Nutrition*, vol. 38, no. 5, pp. 353–380, Jul. 1998, doi: 10.1080/10408699891274246.

[5] E. R. Withell, “The significance of the variation in shape of time-survivor curves,” *J Hyg (Lond)*, vol. 42, no. 2, pp. 124–183, Apr. 1942.

[6] N. Q. Balaban, J. Merrin, R. Chait, L. Kowalik, and S. Leibler, “Bacterial Persistence as a Phenotypic Switch,” *Science*, vol. 305, no. 5690, 2004, Accessed: Sep. 05, 2017. [Online]. Available: http://science.sciencemag.org/content/305/5690/1622.long

[7] Z. Aspridou and K. Koutsoumanis, “Variability in microbial inactivation: From deterministic Bigelow model to probability distribution of single cell inactivation times,” *Food Research International*, vol. 137, p. 109579, Nov. 2020, doi: 10.1016/j.foodres.2020.109579.

[8] G. Kampf, *Antiseptic Stewardship: Biocide Resistance and Clinical Implications*. Springer International Publishing, 2018. doi: 10.1007/978-3-319-98785-9.
